# The involvement of stress granules in aging and aging‐associated diseases

**DOI:** 10.1111/acel.13136

**Published:** 2020-03-14

**Authors:** Xiuling Cao, Xuejiao Jin, Beidong Liu

**Affiliations:** ^1^ State Key Laboratory of Subtropical Silviculture School of Forestry and Biotechnology Zhejiang A&F University Hangzhou China; ^2^ Department of Chemistry and Molecular Biology University of Gothenburg Goteborg Sweden; ^3^ Center for Large‐scale Cell‐based Screening Faculty of Science University of Gothenburg Goteborg Sweden

**Keywords:** aging, aging‐associated diseases, nonmembrane assemblies, proteostasis, RNA‐binding proteins, stress granules

## Abstract

Stress granules (SGs) are nonmembrane assemblies formed in cells in response to stress conditions. SGs mainly contain untranslated mRNA and a variety of proteins. RNAs and scaffold proteins with intrinsically disordered regions or RNA‐binding domains are essential for the assembly of SGs, and multivalent macromolecular interactions among these components are thought to be the driving forces for SG assembly. The SG assembly process includes regulation through post‐translational modification and involvement of the cytoskeletal system. During aging, many intracellular bioprocesses become disrupted by factors such as cellular environmental changes, mitochondrial dysfunction, and decline in the protein quality control system. Such changes could lead to the formation of aberrant SGs, as well as alterations in their maintenance, disassembly, and clearance. These aberrant SGs might in turn promote aging and aging‐associated diseases. In this paper, we first review the latest progress on the molecular mechanisms underlying SG assembly and SG functioning under stress conditions. Then, we provide a detailed discussion of the relevance of SGs to aging and aging‐associated diseases.

## Graphical abstract

Stress granules are sensitive to environmental changes. Age‐dependent cellular changes and breakdown of mitochondrial function and protein quality control system would lead to defects in maintaining normal stress granule assembly, dynamics, disassembly, and clearance. This in turn could promote aging and aging‐associated diseases. In this paper, we provide a detailed discussion of the relevance of stress granules to aging and aging‐associated diseases. And it seems that maintaining a proper stress granule dynamic might be a potential strategy to delay aging and increase lifespan.

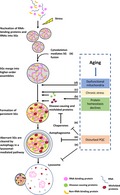

## 1 INTRODUCTION

Cells rely on distinct compartments and organelles, in which molecules are protected against other agents from the surrounding milieu, to concentrate specific cellular components for achieving specific biochemical reactions and biological functions (Aguilera‐Gomez & Rabouille, 2017). Most well‐known organelles are separated from their surroundings by a lipid membrane boundary, for example, the nucleus, mitochondria, endoplasmic reticulum, Golgi apparatus, and peroxisomes. In addition, non‐membrane‐bound cytoplasmic and nuclear compartments have also been identified, and these compartments harbor RNAs and RNA‐binding proteins, including P bodies (Sheth & Parker, 2003), stress granules (SGs) (Arrigo, Suhan, & Welch, 1988; Collier & Schlesinger, 1986), germ granules (Eddy, 1975; Guraya, 1979; Voronina, Seydoux, Sassone‐Corsi, & Nagamori, 2011), neuronal transport granules (Knowles et al., 1996), Cajal bodies (Gall, Bellini, Wu, & Murphy, 1999), and the nucleolus (Brangwynne, Mitchison, & Hyman, 2011; Shaw & Jordan, 1995).

Stress granules are members of this emerging class of membraneless assemblies. They form when cells experience stress conditions, and are thought to influence cellular signaling pathways, and mRNA function, localization, and turn over (Buchan, 2014; Buchan & Parker, 2009; Kedersha, Ivanov, & Anderson, 2013). They present across eukaryotes, including mammalian, plant, and fungal (yeast) cells. In mammalian cells, SGs form in response to heat stress, arsenite exposure, UV irradiation, and viral infection (Kedersha et al., 2013). In plant cells, SGs are induced by hypoxia, high‐salt stress, and oxidative stress, as well as methyl jasmonate, potassium cyanide, and myxothiazol exposure (Gutierrez‐Beltran, Moschou, Smertenko, & Bozhkov, 2015; Nover, Scharf, & Neumann, 1983; Pomeranz et al., 2010; Sorenson & Bailey‐Serres, 2014; Weber, Nover, & Fauth, 2008; Yan, Yan, Wang, Yan, & Han, 2014). SGs form from pools of untranslated mRNA and contain various translation initiation factors, as well as a variety of RNA‐binding proteins and many non‐RNA‐binding proteins (Guzikowski, Chen, & Zid, 2019). SGs are dynamic, complex, and variable assemblies, with composition and structure that can vary dramatically under different types of stresses, such as heat shock, oxidative stress, osmotic stress, nutrient starvation, and UV irradiation. This diversity of SGs supposedly relies on diverse interactions between proteins and RNAs within the SGs and reflects the ability of cells to respond quickly to various environmental stresses (Buchan & Parker, 2009; Protter & Parker, 2016). Recent evidence has revealed that mammalian SGs show liquid‐like behavior while yeast SGs have characteristics similar to those of solid material (Kroschwald et al., 2015). Results from super‐resolution fluorescence microscopy and fluorescence recovery after photobleaching (FRAP) experiments reveal that SGs have two distinct layers with different components, functions, and dynamics: a stable inner core structure surrounded by a less dense shell layer. The components in the core structure are believed to be less dynamic, while the components in the shell layer are more dynamic. Two models were proposed for SG assembly. The first is that SG assembly is initiated by formation of a stable core containing a diverse proteome, followed by rapid growth of this core. Subsequently, these initial small granules merge to form larger mature stress granules, through liquid–liquid phase separation (LLPS). Alternatively, LLPS of translationally repressed ribonucleoproteins occurs first, and then, high concentrations of proteins in phase‐separated droplets promote the formation of the core (Jain et al., 2016; Wheeler, Matheny, Jain, Abrisch, & Parker, 2016).

## 2 STRESS GRANULE FORMATION AND REGULATION

Unlike cellular compartments surrounded by lipid bilayer membranes, which physically separate the interior and exterior of the compartments, SGs lack a physical barrier to separate their components from the surrounding medium. For many years, it remained elusive how molecules could be clustered into a discrete area without a surrounding barrier, and how such clustered molecules could modulate their structures and internal biochemical activities. Increasing evidence indicates that LLPS driven by multivalent weak macromolecular interactions (protein–protein, protein–RNA, and RNA‐RNA interactions) is an important organizing principle underlying the self‐assembly of membraneless compartments (Gomes & Shorter, 2019; Hyman, Weber, & Julicher, 2014). LLPS is a process in which a well‐mixed liquid solution separates into two distinct liquid phases. One phase is enriched in some certain components, and the other is depleted of these components (Alberti & Dormann, 2019). In recent years, an increasing body of evidence has indicated that LLPS plays an important role in cellular organization and the assembly of membraneless organelles, which are comprised of highly concentrated proteins and RNAs (Alberti & Dormann, 2019). In contrast to membrane‐bound organelles, which utilize active transport of molecules across the surrounding membranes to maintain their compositions, the liquid state of the two LLPS phases ensures that the components within these two phases can easily rearrange and can quickly exchange materials with each other. This allows liquid non‐membrane‐bound compartments such as SGs to remain separate from the liquid cytoplasm but able to rapidly respond to environmental stresses. SGs can accomplish this rapid response through entry of macromolecules into the SGs or release of certain components from the SGs (Alberti & Dormann, 2019; Hyman et al., 2014). However, evidence indicates that this process is sensitive to cellular environmental changes such as temperature, pH, and even concentration of molecules (Alberti & Hyman, 2016). High concentrations of macromolecules must reach a critical threshold to start LLPS. However, if a certain concentration is exceeded, macromolecules within the SGs, such as proteins, will tend to form aggregates. This can lead the LLPS‐derived liquid assembly to take on gel‐like or solid‐like properties, which may not function properly (Alberti & Dormann, 2019). Such pathological assemblies, induced by LLPS, are involved in the onset of diseases including amyotrophic lateral sclerosis (ALS), Alzheimer's disease, and Parkinson's disease (Alberti & Dormann, 2019; Falahati & Haji‐Akbari, 2019). The molecules that undergo phase separation include proteins composed of modular interaction domains and intrinsically disordered regions (IDRs). RNA and DNA molecules that harbor multiple interaction regions for binding to other proteins or nucleic acids can also undergo phase separation independently or synergistically with proteins (Jain & Vale, 2017; Molliex et al., 2015). Different kinds of molecular interactions between the diverse regions discussed above can promote LLPS. We have summarized these interactions that promote LLPS and SG formation in Table 1. In the following sections, we mainly focus on the molecular mechanisms underlying SG assembly and discuss the essential factors responsible for the regulation of SG assembly.

**Table 1:** Molecular interactions that promote liquid–liquid phase separation and SG formation

Interaction	Property	Example	References
Protein–protein interaction			
SLiM interactions	Usually regulate low‐affinity interactions, easily modulated by PTM	Decapping complexes	Jonas and Izaurralde (2013)
Kinked cross‐β‐sheets between IDRs	Interact weakly through polar atoms and aromatic side chains	FUS, hnRNPA1, nup98	Hughes et al. (2018)
Interactions between RGG/RG motifs and PrLDs	Cation–π interactions	FUS	Bogaert et al. (2018), Qamar et al. (2018), Wang, Choi, et al. (2018)
Interactions between RNA‐binding domain and IDR	Tyrosine–arginine interactions (different with generic cation–π interactions)	FET family proteins	Wang, Choi, et al. (2018)
Polymerization by oligomerization domains		TDP‐43	Wang, Conicella, et al. (2018)
Protein–RNA interaction			
Interactions between RGG/RG motifs and RNA	Hydrogen bonding, π‐stacking, and cation–π interactions	EWS, FUS, FMRP, …	Chong et al. (2018)
Interactions between cationic peptides with RNA	Charge–charge neutralization		Aumiller and Keating (2016)
Interactions between RNA‐binding domain and RNA		FUS, Whi3, …	Burke, Janke, Rhine, and Fawzi (2015), Zhang et al. (2015)
RNA‐RNA interaction			
Self‐assembly of RNA	Watson–Crick and non‐Watson–Crick interactions between bases, base stacking		Van Treeck and Parker (2018)

### 2.1 Roles of proteins with intrinsically disordered regions in SG formation

Multiple high‐content studies have generated detailed lists of SG components in both yeast and mammalian cells (Youn et al., 2019). Here, we summarize the results of these works in Table 2 to provide an overview of all the SG components identified together with the corresponding large‐scale experimental methods applied. Only a fraction of these are thought to be necessary for the formation and maintenance of SGs under stress conditions. Proteins that are essential and sufficient to drive the formation of SGs are called scaffold proteins. In the absence of these scaffold proteins, the compartments do not form or are unstable (Ditlev, Case, & Rosen, 2018). For example, certain translation repressors, such as caprin‐1 and TIA‐1, RNA‐binding proteins, like G3BP, and enzymes with ATPase activities, have been shown to function as scaffold proteins (Buchan & Parker, 2009; Gilks et al., 2004; Jain et al., 2016; Kedersha et al., 2016; Tourriere et al., 2003). These proteins can interact with RNAs and recruit other client proteins to the SGs to form a promiscuous interaction network. They are essential for the formation of SGs.

**Table 2:** SG components identified from large‐scale studies

Model system	Stress	Experimental approaches	Number of hits	References
Proteins				
Yeast, mammalian cell	NaN_3_ (yeast), NaAsO_2_ (mammalian)	Centrifugation and immunoprecipitation, and mass spectrometry	Yeast: 228 components; Mammal: 317 components	Jain et al. (2016)
Mammalian cell	NaAsO_2_, heat shock	Ascorbate peroxidase (APEX) proximity labeling, mass spectrometry, and immunofluorescence	~150 components	Markmiller et al. (2018)
Mammalian cell	Arsenite	Proximity‐dependent biotinylation techniques and mass spectrometry	106 components	Youn et al. (2018)
Mammalian cell	Arsenite	RNA‐mediated interference‐based screen	101 regulators, 9 components	Ohn et al. (2008)
Yeast		Nonessential gene deletion mutants and fluorescence microscopy	101 regulators, 2 components	Buchan et al. (2013)
Yeast	Glucose starvation	mRNA‐binding protein screening and fluorescence microscopy	10 components	Mitchell, Jain, She, and Parker (2013)
RNAs				
Yeast, mammalian cell	Arsenite	RNA sequencing analysis and single‐molecule fluorescence in situ hybridization validation	Yeast: 916 mRNA and some ncRNAs; Mammal: 10% total mRNAs and 0.6% total ncRNAs	Khong et al. (2017)

Multivalent interactions among protein intrinsically disordered regions or intrinsically disordered proteins (IDPs), which contain low‐complexity domains (LCDs) or prion‐like domains (PrLDs), are major contributors to protein phase separation and SG formation. These low‐complexity sequences have low amino acid diversity (Gomes & Shorter, 2019) and can be identified by their similarity in amino acid composition to the known human and yeast prions (Gilks et al., 2004). Many RNA‐binding proteins found in SGs are characteristically composed of these sequences, which have been shown to be important for SG formation and targeting. Examples of proteins with prion‐like domains that are responsible for granule assembly include T‐cell intracellular antigen‐1 (TIA‐1) and fused in sarcoma (FUS). The prion‐like domain of TIA‐1 is essential for its recruitment to SGs and is also necessary for promotion of SG formation. Further, the function of the TIA‐1 PrLD can functionally substitute with the PrLD of the yeast Sup35 protein (Gilks et al., 2004). The RNA‐binding protein FUS localizes to SGs through its N‐terminal IDR, and mutations in this region prevent SG accumulation (Kato et al., 2012).

Recent studies have pointed out the importance of promiscuous interactions caused by these prion‐related, disordered regions. Through such interactions, the proteins are able to act cooperatively in promoting LLPS (Van Treeck & Parker, 2018). For example, short linear motifs (SLiMs) within IDRs can interact with the surface of other well‐folded protein domains (Jonas & Izaurralde, 2013). Also, interactions between IDRs might form cross‐strand beta‐zippers to stabilize granules (Protter & Parker, 2016). Recently, a new type of beta‐sheets has been identified, namely kinked cross‐beta‐sheets or low‐complexity aromatic‐rich kinked segments (LARKS). Unlike classic steric zippers of amyloid fibrils, LARKS are characterized by relatively weak stability. These transient cross‐beta‐contacts might also contribute to LLPS (Hughes et al., 2018). RGG/RG motifs are segments that occur in low‐complexity disordered regions and have a high affinity for RNA. Thus, multivalent interactions between RGG motifs and RNAs are thought to underlie phase separation in the formation of SGs (Chong, Vernon, & Forman‐Kay, 2018; Gomes & Shorter, 2019). Recent evidence has also indicated that intracellular interactions between tyrosine residues from PrLDs and arginine residues in RGG motifs could drive phase separation (Bogaert et al., 2018; Qamar et al., 2018; Wang, Choi, et al., 2018; Yoshizawa et al., 2018). Besides these interactions, IDRs could also work together with RNA recognition motifs (RRMs) to modulate phase behavior synergistically. For example, FUS phase separation is governed by interactions between tyrosine residues in a PrLD and arginine residues in an RNA‐binding domain (Wang, Choi, et al., 2018). These domains could form diverse weak interactions such as π‐π interactions, cation–π interactions, charge–charge interactions, and intermolecular cross‐β‐contacts, which are all thought to be important drivers of phase separation (Gomes & Shorter, 2019).

### 2.2 RNAs are involved in stress granule assembly

Stress granule formation requires a pool of RNA molecules that can bind to the RNA‐binding domains of numerous proteins to form multivalent RNA–protein interactions and contribute to the formation of higher‐order assemblies. In addition, RNA can influence other aspects of SG assembly. First, SG formation begins with inhibition of translation initiation and polysome disassembly; adding of drugs that trap mRNAs in polysomes will reduce SG assembly (Buchan, Muhlrad, & Parker, 2008; Kedersha et al., 2000; Kedersha, Gupta, Li, Miller, & Anderson, 1999). Second, RNAs critically modulate phase separation behavior of some prion‐like proteins. High RNA/protein ratios prevent phase separation, while low ratios have an opposite effect. For example, low RNA levels lead to excessive phase separation of the SG components, FUS and TAR DNA‐binding protein‐43 (TDP‐43), in the cytoplasm. Thus, RNA concentration‐dependent phase separation can modulate the alteration of RNA‐binding proteins from a dynamic state to a pathological solid state (Maharana et al., 2018). Finally, some evidence indicates that intermolecular RNA‐RNA interactions and RNA structure play a role in forming and determining the composition of certain RNP granules. For example, protein‐free yeast RNAs are capable of self‐assembly in vitro under physiologically relevant conditions and could define the stress granule transcriptome (Van Treeck et al., 2018). Additional observations have revealed that delivery of exogenous mRNA and ssDNA to the cytoplasm could trigger the formation of SGs, possibly driven by RNA‐RNA and DNA‐RNA interactions (Bounedjah et al., 2014; Mahadevan et al., 2013). Expansion of hexanucleotide GGGGCC (G4C2) repeats in noncoding regions of the chromosome 9 open reading frame 72 (*C9ORF72*) is the most common mutation associated with amyotrophic lateral sclerosis and frontotemporal dementia (C9‐ALS/FTD) (DeJesus‐Hernandez et al., 2011; Renton et al., 2011). Transfection of RNA G4C2 (rG4C2) could promote SG assembly in a repeat length‐ and G‐quadruplex structure‐dependent manner, indicating essential roles for RNA and RNA structure in SG assembly (Fay, Anderson, & Ivanov, 2017). Similarly, Langdon and colleagues observed that the secondary structure of mRNA determines the molecular composition of membraneless compartments (Langdon et al., 2018). Thus, for assemblies like SGs, which contain a high local concentration of RNA molecules, RNA‐RNA interactions and RNA structure seem to be general features of importance for their formation. Detailed information about the roles of RNA in forming SGs and other RNP granules can be found in recent reviews (Garcia‐Jove Navarro et al., 2019; Van Treeck & Parker, 2018).

### 2.3 Protein post‐translational modification and the cytoskeleton system regulate stress granule formation

Protein post‐translational modification (PTM) is another mechanism by which cells control the localization of SG components and assembly/disassembly of SGs (Buchan & Parker, 2009). Researchers have shown that SGs are enriched in proteins that have undergone PTM, including phosphorylation, dephosphorylation, methylation, neddylation, acetylation, *O*‐GlcNAc, SUMOylation, and poly(ADP‐ribosylation) (see Table 3 for a full list of currently known PTMs influencing SG dynamics). For example, hypophosphorylated Grb7 can be recruited by Hu antigen R (HuR) to SGs to stabilize SG formation under stress. Conversely, phosphorylation of Grb7 facilitates SG disassembly during recovery through dissociation of Grb7 from HuR and other SG components (Tsai, Ho, & Wei, 2008). Previous work also showed that phosphorylation of Ras‐GTPase‐activating protein SH3 domain‐binding protein 1 (G3BP1) on Ser 149, a key player in SG assembly, impairs G3BP1 self‐association and inhibits arsenite‐induced stress granule assembly (Tourriere et al., 2003). However, a recent study showed that G3BP1‐S149 is indeed phosphorylated, but S149 phosphorylation does not change upon stress treatment and therefore is not a simple switch that regulates SGs. The phenotype of the original S149E mutant emerged because of an accidental S99P mutation. Despite this, Panas et al. pointed out that phosphorylation of S149 could not be excluded from influencing other processes related to SG or signaling, which is regulated by interactions between G3BP1 and other proteins (Panas et al., 2019). Thus, further investigation is needed to clarify the role of phosphorylation in SG regulation. In addition, evidence indicates that methylation of arginine at an RGG motif, the preferred site for methylation, in some RNA‐binding proteins is necessary for both themselves and Tudor domain‐containing protein recruitment to SGs (De Leeuw et al., 2007; Dolzhanskaya, Merz, Aletta, & Denman, 2006; Goulet, Boisvenue, Mokas, Mazroui, & Cote, 2008). NEDD8 (neural precursor cell‐expressed developmentally downregulated protein 8) is a small, ubiquitin‐like protein that covalently attaches to protein substrates in a manner similar to ubiquitination, known as neddylation. Neddylation of serine/arginine (SR)‐rich splicing factor 3 (SRSF3), an SG regulator, is necessary for SG formation (Jayabalan et al., 2016). Reversible protein acetylation is another important protein PTM modulated by histone acetylases and histone deacetylases. Histone deacetylase 6 (HDAC6), a cytoplasmic deacetylase, exhibits deacetylase activity that is important for regulation of SG formation, indicating a new role for acetylation in the stress response (Kwon, Zhang, & Matthias, 2007). Based on RNA‐mediated interference screening, some genes that participate in reversible O‐linked N‐acetylglucosamine (*O*‐GlcNAc) modification were found to be important for SG assembly. Further, the substrates of *O*‐GlcNAc modification might be major components of SGs (Ohn, Kedersha, Hickman, Tisdale, & Anderson, 2008). SUMO is a small ubiquitin‐like molecule that is covalently attached to target proteins to regulate protein–protein interactions, protein localization, and protein function (Hannoun, Greenhough, Jaffray, Hay, & Hay, 2010). SUMOylation has also been reported to play a role in recruitment of proteins to SGs, since SG assembly is impaired when SUMOylation of eukaryotic initiation factor eIF4A2 is disabled (Jongjitwimol, Baldock, Morley, & Watts, 2016).

**Table 3:** Post‐translational modifications and their functions in stress granule regulation

Protein	Species	Modification	Position	Function	References
Grb7	Mammalian cell	Phosphorylation	—	Facilitation of SG disassembly during recovery	Tsai et al. (2008)
G3BP1	Mammalian cell	Phosphorylation	—	Regulation of SG assembly？	Tourriere et al. (2003), Panas et al. (2019)
TTP	Mammalian cell	Phosphorylation	Ser52, Ser178	Exclusion of TTP from SG, leading to separation of SG from PB	Stoecklin et al. (2004)
MEX3B	Mammalian cell	Phosphorylation	Ser462	Promotion of SG‐PB fusion	Courchet et al. (2008)
G3BP1	Mammalian cell	Methylation	RGG domain	Repression of SG assembly	Tsai et al. (2016)
CIRP	Mammalian cell	Methylation	RGG domain	Recruitment into the SG	De Leeuw et al. (2007)
FMRP	Mammalian cell	Methylation	RGG domain	Recruitment into the SG	Dolzhanskaya et al. (2006)
TDRD3‐interacting proteins	Mammalian cell	Methylation	RG‐rich motif	Recruitment of TDRD3 into the SG	Goulet et al. (2008)
SRSF3	Mammalian cell	Neddylation	Lys85	Promotion of SG assembly	Jayabalan et al. (2016)
—	Mammalian cell	Deacetylation	—	Promotion of SG assembly	Kwon et al. (2007), Jedrusik‐Bode et al. (2013)
RACK1, Prohibitin‐2, RPS3, RPL13a	Mammalian cell	*O*‐GlcNAc	—	Promotion of SG assembly	Ohn et al. (2008)
eIF4A2	Mammalian cell	SUMOylation	K226	Promotion of SG formation	Jongjitwimol et al. (2016)
—	Mammalian cell	Poly (ADP) ribosylation	—	Regulation of SG assembly	Leung et al. (2011)

Note

—, data not available.

Recently, another PTM, poly(ADP‐ribosylation) (PARylation), has received increasing interest due to the role of PARylation in SG regulation and pathogenesis of neurodegenerative diseases, as well as the therapeutic value of poly(ADP‐ribose) polymerase (PARP) inhibition in treatment of neuropathies (Grimaldi et al., 2019). Poly(ADP‐ribose), or pADPr, a macromolecule synthesized by PARPs, regulates many physiological processes in the nucleus (Schreiber, Dantzer, Ame, & Murcia, 2006). Recent studies have found that PARylation level can serve as a major regulator in the assembly and disassembly of SGs. Specific PARPs and pADPr glycohydrolases (PARGs) are localized to cytoplasmic SGs (Leung et al., 2011). Overexpression of PARPs can induce the formation of SG without stress, and overexpression of PARGs can prevent SG formation. Also, a high level of PARylation in the cell delays disassembly of SGs (Catara et al., 2017; Leung, 2014; Leung et al., 2011). An additional study found that the formation of stress granules is a strategy adopted by cancer cells to resist chemo/radiotherapy. Although the role of PARylation in SG formation in cancer cells is unclear, it can be speculated that inhibition of SGs through inhibition of PARylation may be beneficial in the treatment of tumor resistance (Grimaldi et al., 2019).

Disease‐associated, aggregation‐prone RNA‐binding proteins (RBPs) such as heterogeneous nuclear ribonucleoprotein A1 (hnRNP A1) and TDP‐43 are predominantly nuclear proteins and are typical components of RNP granules. Excessive cytoplasmic localization would impair their functions in the nucleus and induce cytotoxicity (Kim et al., 2013; Neumann et al., 2006). Evidence indicates that PARylation levels can modulate RBPs’ cytoplasmic translocation and SG localization, protein–protein interaction, as well as phase separation behaviors (Duan et al., 2019). Inhibition of PARylation significantly suppresses the cytotoxicity of hnRNP A1 and TDP‐43 in motor neuron‐like NSC‐34 cells. It can also suppress TDP‐43 overexpression‐mediated neurodegeneration in a *Drosophila* ALS model through downregulation of PARP and upregulation of PARG. These results indicate the potential therapeutic value of PARylation regulation in the treatment of aging‐related neurodegenerative diseases (Duan et al., 2019). The importance of PARP in neuropathy is also demonstrated by the fact that generation of PAR accelerates pathologic α‐synuclein fibrillization and cell death, and thus, genetic or pharmacological inhibition of PARP could mitigate pathologic α‐syn toxicity (Kam et al., 2018). PARP inhibitors have already been used for cancer treatment (Berger et al., 2018; Jain & Patel, 2019). Based on the above‐mentioned examples regarding the involvement of PARP in SG dynamics and neuropathy, the development of PARP inhibitors for aging‐related neurodegeneration treatment could also yield promising advances.

**Figure 1:**
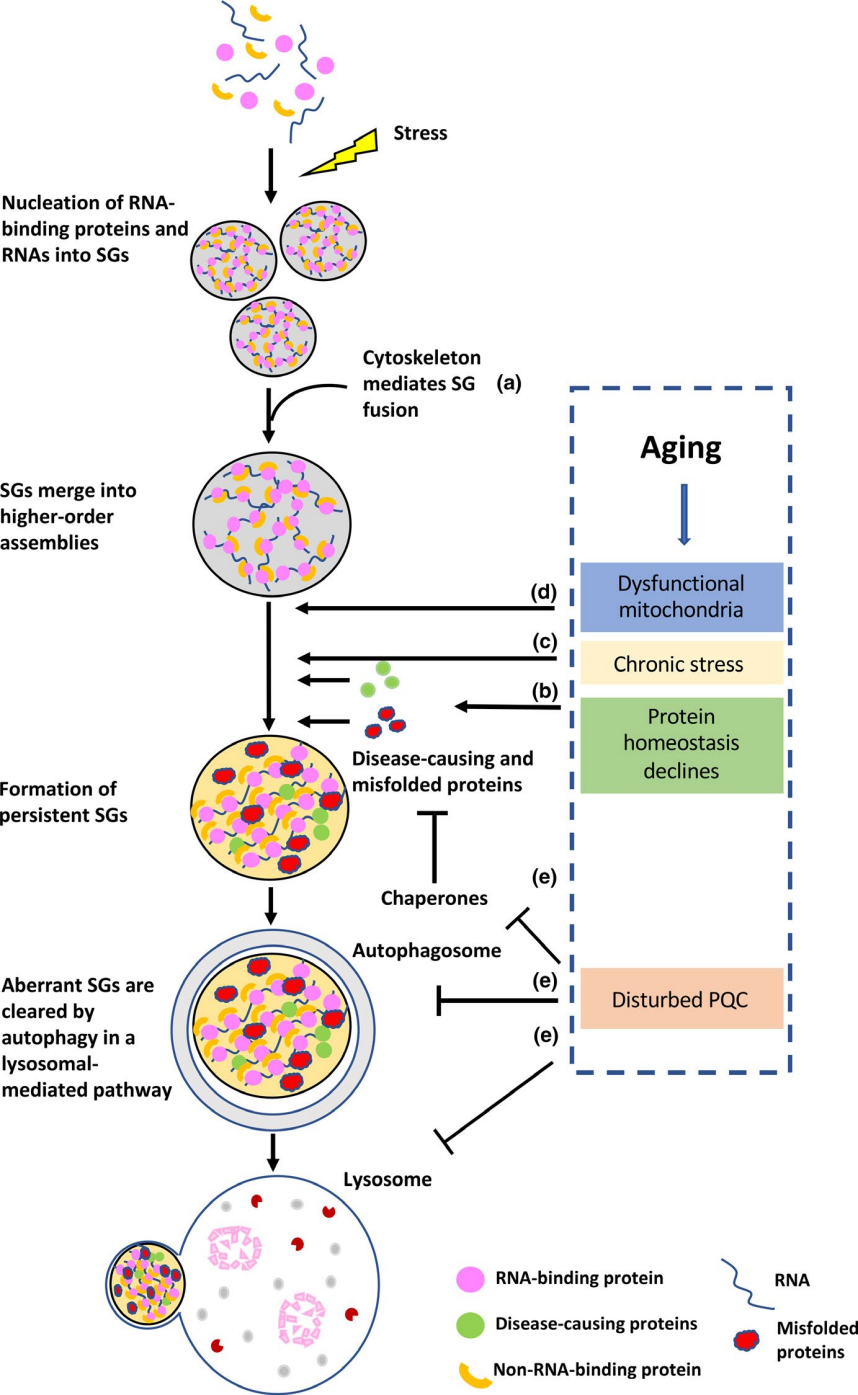
Effects of aging on SG assembly, dynamics, and clearance. Formation of SGs begins with nucleation of various RNA‐binding proteins and RNAs. The SGs then grow into larger assemblies via additional protein–protein and protein–RNA interactions. These complexes coalesce into higher‐order SGs in a cytoskeleton system‐dependent manner (a). Aging‐associated mitochondrial dysfunction and inactive metabolism might lead to limited control of this process and aberrant SGs (d). In addition, aging‐associated disease‐causing proteins, misfolded proteins caused by protein homeostasis decline (b), and other chronic stress (c) during aging lead to impaired SG dynamics and persistent SGs. Aberrant SGs can be cleared by autophagy under normal conditions, but with age, disturbed PQC can have a negative effect on the removal of aberrant SGs (e)

Besides the above‐mentioned PTMs, the cytoskeletal system is another factor involved in the regulation of SG formation (Kwon et al., 2007; Loschi, Leishman, Berardone, & Boccaccio, 2009). SGs are dynamic structures that exchange materials with other granules and/or with their surroundings. The cytoskeleton system is thought to be a scaffold for SG assembly and dynamic maintenance, as well as movement, fusion, and fission (Figure 1a). For example, microtubules and motor proteins are critical for SG fusion and disassembly, as evidenced by the finding that drugs that disrupt microtubules and inhibit motor proteins reduce the appearance of SGs (Kwon et al., 2007). In addition, the motor proteins kinesin and dynein are localized in SGs and regulate SG assembly and disassembly (Loschi et al., 2009).

## 3 FUNCTIONS OF STRESS GRANULES

Cells are frequently exposed to fluctuating, potentially adverse environmental conditions. Thus, formation of SGs allows cells to adapt to diverse environmental changes and provides protection for key cellular components. This is reflected in some SG‐defective mutants, which are more sensitive to stress. These include HDAC6‐deficient mouse embryo fibroblasts, p90 ribosomal S6 kinase family Ser/Thr kinase (RSK2)‐silenced cells, and yeast poly(A)‐binding protein (Pab1) mutants (Eisinger‐Mathason et al., 2008; Kwon et al., 2007; Riback et al., 2017; Yang, Shen, et al., 2014). So, how do SGs help cells to survive under stress? First, as SGs contain a high local concentration of diverse proteins and mRNAs, the efficiency of certain reaction kinetics may be increased by close contact within a confined space. For example, concentration of translation factors and mRNAs together in SGs might promote their interaction and thereby enhance the formation of translation initiation complexes (Buchan et al., 2008). It is worth noting, however, that sequestration of resident chemical species does not always result in an acceleration of all reaction rates. The highly concentrated scaffolds and clients within biomolecular condensates may interface with each other, leading to reduced enzyme activities and disrupted interactions between the scaffold and other proteins (Banani, Lee, Hyman, & Rosen, 2017).

The second role of SGs is signal transduction in response to cellular stress. Some specific factors that are sequestered in SGs will alter the regulation of particular signaling pathways, in which these factors participate (Kedersha et al., 2013). For example, target of rapamycin complex 1 (TORC1) signaling can be modulated by SGs upon heat stress: TORC1 signaling is blunted by sequestering TORC1 in SGs, and signaling is reactivated upon SG disassembly (Takahara & Maeda, 2012). Additionally, astrin‐induced localization of mTORC1 components in SGs suppresses oxidative stress‐induced apoptosis (Thedieck et al., 2013). Moreover, SG formation can negatively regulate the stress‐activated p38 and JNK MAPK (SAPK) pathways that trigger the apoptotic response. This is accomplished through sequestration of the receptor for activated C kinase (RACK1) in the SG, thus protecting the cell from death (Arimoto, Fukuda, Imajoh‐Ohmi, Saito, & Takekawa, 2008). Furthermore, ribosomal subunit eIF4G can interact with and recruit TNF‐α receptor‐associated factor 2 (TRAF2) to SGs when a cell is confronted with stress, thus inhibiting tumor necrosis factor signaling by lowering TRAF2 biological activity in the cytoplasm (Kim, Back, Kim, Ryu, & Jang, 2005). Plasminogen activator inhibitor‐1 (PAI‐1), an activator of senescence, is also localized to SGs in senescent cells. This indicates that SGs can counteract senescence by recruiting a key factor to the SGs and disrupting its senescence function (Omer et al., 2018).

The third function of SG is a protective one: Formation of SGs allows cells to "bounce back" and thrive once the stress subsides. SGs do this by temporarily storing and protecting mRNAs and proteins from autophagy and degradation by proteasomes throughout the duration of the stress, allowing a rapid restart of translation and other signaling pathways upon release from the stress (Guzikowski et al., 2019). Yeast poly(A)‐binding protein (Pab1), a marker of SGs, can act as a stress sensor and undergo a phase transition to a hydrogel to protect stressed cells and then to help cells better recover upon stress relief (Riback et al., 2017). Results have also shown that the yeast pyruvate kinase protein Cdc19 can form reversible aggregates that co‐localize with SGs during stress, which is a protective mechanism from stress‐induced degradation. Upon cessation of stress and resumption of growth, it allows quick re‐entry of Cdc19 into the cell cycle without re‐expression (Saad et al., 2017).

## 4 RELATIONSHIP BETWEEN STRESS GRANULES, AGING, AND AGING‐RELATED DISEASES

### 4.1 Aging and aging‐related diseases

Aging is a ubiquitous, progressive, and usually irreversible biological process accompanied by a decline in physiological and reproductive functions. This process normally begins shortly after the formation of the fertilized egg and spans the whole lifespan of the body (Veitia, Govindaraju, Bottani, & Birchler, 2017). It has been less than 40 years since aging research entered the era of modern molecular biology with isolation of the first long‐lived nematode mutant (Klass, 1983). Nowadays, research on aging has led to a deeper understanding of both aging itself and its relationship with various diseases. Nine tentative hallmarks have been listed in a recent review to represent common denominators of aging in different organisms: genomic instability, telomere attrition, epigenetic alterations, loss of proteostasis, deregulated nutrient sensing, mitochondrial dysfunction, cellular senescence, stem cell exhaustion, and altered intercellular communication (Lopez‐Otin, Blasco, Partridge, Serrano, & Kroemer, 2013).

Aging is a predominant risk factor for many disorders, including cancer, neurodegenerative diseases, and cardiovascular diseases. Aging itself is not a disease, but aging and aging‐related diseases sometimes share the same basic molecular and cellular mechanisms (Franceschi et al., 2018). For example, as aging progresses, the ability of cells to maintain protein homeostasis declines, leading to the formation of widespread intracellular protein aggregates (Taylor & Dillin, 2011). In addition, neurodegenerative diseases such as Alzheimer's, Parkinson's, and Huntington's diseases are characterized by the presence of one or more protein aggregates in the cells at the site of the disease. Although the proteins that form aggregates in these diseases are different, and some are cytotoxic, the disease onset for all of them is during middle and old age. Emerging evidence indicates that enhanced regulation of protein balance can reduce the toxicity of protein aggregates in degenerative diseases (Gao et al., 2015; Jayaraj, Hipp, & Hartl, 2020; Labbadia & Morimoto, 2015), supporting the connection between aging‐associated proteostasis decline and degenerative diseases.

### 4.2 Aberrant stress granules: a driver of aging and aging‐related diseases?

Age‐associated proteostasis disruption is characterized by the appearance of various physiological and pathological protein aggregates in various tissues. In different models, hundreds of proteins are highly prone to form aggregates during aging (Ayyadevara et al., 2016; David et al., 2010; Demontis & Perrimon, 2010; Reis‐Rodrigues et al., 2012; Tanase et al., 2016). Proteomic analysis of aggregations associated with aging in *C. elegans* highlights the presence of certain RNA granule components in wild‐type animals that are absent in long‐lived mutants (Lechler et al., 2017), implying that RNA granules, or at least certain compositions of RNA granules, become less dynamic as aging progresses. The absence of certain RNA granule components in aggregations in long‐lived animals indicates the relevance of RNA granule dynamics to longevity (Lechler et al., 2017). More importantly, the classic SG proteins PAB‐1 and TIAR‐2 can form aggregates in aged *C. elegans,* and a high level of aggregation of these SG components is associated with smaller animal size, reduced fitness, and shorter lifespan (Lechler et al., 2017). This suggests that SG protein aggregation might accelerate aging and reduce lifespan.

LCDs of RNA‐binding proteins (RBPs) play an important role in SG formation because these motifs allow multiple interactions; along with their flexibility in folding, this allows the formation of a dense protein–protein and protein–RNA interaction network (Buchan, 2014; Guzikowski et al., 2019). However, their conformational flexibility also leads these proteins to be aggregation‐prone in general; they can undergo spontaneous transitions into an aggregated solid state, which can act as a seed to incorporate other aggregation‐prone proteins to form irreversible aggregations (Alberti, Halfmann, King, Kapila, & Lindquist, 2009). Some mutant forms of these RBPs, especially those with mutations in their prion‐like domain, form aggregates more easily. These aberrant, irreversible aggregations could lead to persistent SGs after the removal of the stress, which would further cause other pathological changes within the cell. This is consistent with the fact that aberrant RBP aggregation is a hallmark of numerous neurodegenerative disorders (Arai et al., 2006; Neumann et al., 2006, 2009).

In addition to RBP mutants and RBP‐including aggregates within SGs, which can disturb SG dynamics, some proteins can form aggregates independently from SG formation. They can aggregate in association with lipid membranes such as mitochondrial membranes and endoplasmic reticulum, which can lead to dysfunction of these membrane‐bound organelles (Hebda & Miranker, 2009; Stefani, 2010; Zhou et al., 2014). Also, some cytosolic amyloid‐like protein aggregates such as TDP‐43 have been shown to disturb nuclear integrity and nucleocytoplasmic transport by sequestering nucleocytoplasmic transporters (Gasset‐Rosa et al., 2017; Woerner et al., 2016). These effects can potentially lead to accelerated aging and onset of neurodegenerative diseases.

Abnormal accumulation of misfolded proteins that form insoluble fibrillar aggregates is thought to be one of the major causes of many neurodegenerative diseases (Furukawa & Nukina, 2013). Although fibrils are toxic to some degree, numerous studies suggest that protein oligomers are more strongly correlated with disease severity than fibrils in neurodegenerative diseases (Erten‐Lyons et al., 2009; Guerrero‐Munoz, Castillo‐Carranza, & Kayed, 2014; Lasagna‐Reeves et al., 2011; Wu et al., 2013). For instance, amyloid‐β oligomeric aggregates can induce cognitive deficits in rodent models (Sengupta, Nilson, & Kayed, 2016) and increased levels of tau oligomers are correlated with the onset of clinical symptoms and are the cause of disease propagation (Gerson et al., 2014; Lasagna‐Reeves et al., 2012). The toxicity of oligomeric aggregates may be due to their ability to seed the aggregation of other normally folded monomeric proteins, sequester cell membranes or organelles, and be more effectively transported from initially affected brain regions to other regions, compared with fibrillar aggregates (Langer et al., 2011; Silveira et al., 2005). In addition, although specific amyloid fibrils lead to cytopathology in neurodegenerative diseases (Bauerlein et al., 2017; Ramdzan et al., 2017; Woerner et al., 2016), growing evidence suggests that fibrillar aggregates have positive functions under certain conditions of environmental stress (Farmer, Gerson, & Kayed, 2016; Furukawa & Nukina, 2013). For example, yeast prion Sup35 can form amyloid‐like aggregates in vitro and i*n vivo*. Upon formation of fibrils, Sup35 would lose its function as translation terminator and confer cell a survival advantage under oxidative stress (True, Berlin, & Lindquist, 2004). In animals, amyloid formation has been proposed to modulate signal transduction and clear misfolded proteins (Furukawa & Nukina, 2013; Li et al., 2012). Thus, the complexity of the potential consequences of protein aggregation on aging and diseases, as well as direct causal link between aggregation and neurotoxicity, requires further investigation.

### 4.3 How do aging and aging‐related diseases affect stress granules?

Emerging evidence suggests that LLPS‐driven SG assembly is associated with cancer, virus infections, and age‐related neurodegenerative disorders (Mahboubi & Stochaj, 2017). A recent study found that SG components were mislocalized from the nucleus to the cytoplasm in aged animals when they were exposed to mild stressful conditions, but no mislocalization occurred in young animals under the same conditions (Lechler & David, 2017). It is well shown that during aging, cellular surveillance systems are disturbed, resulting in the aberrant cytoplasmic localization of nuclear RBP. We will therefore discuss how stress granules might be influenced by aging and aging‐related diseases in detail below.

#### 4.3.1 The relationship between cellular senescence state and SG formation

Cellular senescence is an irreversible cell cycle arrest state. The number of cells entering this state increases with aging, and it has been widely assumed that cellular senescence contributes to aging (Lopez‐Otin et al., 2013). Cellular senescence has been found to impair the proper formation of both canonical and noncanonical SGs in kidney cells (Moujaber et al., 2017). Canonical SG formation is reduced by the depletion of transcription factor Sp1, which regulates the abundance of the SG‐nucleating proteins G3BP1 and TIA‐1/TIAR. In addition, senescence can cause translation initiation factor eIF2α hyperphosphorylation and the loss of CreP, which also correlates with the aging‐related hyperphosphorylation of eIF2α (Moujaber et al., 2017). These results indicate that when cells enter the senescence state, two essential SG proteins, eIF2α and the transcription factor Sp1, can work as direct aging‐related targets, causing significant deficiencies in SG production. Similarly, a recent study found that constitutive exposure to stress could induce the formation of SGs in proliferating cells, but not in fully senescent human fibroblasts (Omer et al., 2018). It has also been reported that the formation of SGs during early stages of senescence is sufficient to prevent senescence. This process involves the recruitment of PAI‐1 to the SGs. Recruitment of PAI‐1 to SGs interferes with secretion of PAI‐1 and, consequently, cytoplasmic retention of cyclin D1, which further promotes the phosphorylation of pRB, leading to the prevention of cell cycle arrest (Omer et al., 2018). Thus, as SGs can regulate genes that play important roles in senescence, SGs could function as a target of intervention to modulate the senescence process.

However, senescent cells under acute stress may behave differently from cells under chronic stress. Lian et al. found that senescent cells are able to form SGs under acute stress, and the number of SGs significantly increases, compared with nonsenescent cells (Lian & Gallouzi, 2009). This increase correlates with a rapid decrease in the expression levels of the senescence‐associated gene, p21 (Lian & Gallouzi, 2009), which is not found in senescent cells under chronic and persistent stress (Omer et al., 2018). This suggests that how senescence process and the behavior of senescent cells respond to stress might depend on the method used to induce stress and whether or not the stress is physiologically relevant. At the same time, Lian et al. found that, accompanied by the formation of increased number of SGs under acute stress, the disassembly of SGs is also delayed when cells are fully senescent upon acute stress removal. As a consequence of this, translation and synthesis of some vital proteins in fully senescent cells would occur at a slower rate than in proliferative cells after stress removal, affecting normal cellular processes (Lian & Gallouzi, 2009). Thus, we can see that although senescent cells respond differently to chronic stress and acute insult, their ability to regulate SGs and adaptability to stress are impaired in general. This could in part explain the well‐known phenomenon that senescent cells recover from stress more slowly than nonsenescent cells; it could also help to explain their high correlation with age‐related diseases (Gallouzi, 2009; Honda & Matsuo, 1987; Rosenfeldt et al., 2004; Zarzhevsky, Menashe, Carmeli, Stein, & Reznick, 2001).

#### 4.3.2 Aging‐induced cellular environmental and metabolic changes affect stress granule dynamics

The process of aging is linked with the decline of multiple cellular events, including loss of protein homeostasis, decreased vacuolar acidity, and increased reactive oxygen species (ROS) damage (Denoth Lippuner, Julou, & Barral, 2014; Lopez‐Otin et al., 2013). Such intracellular environmental changes during aging can further affect SG formation. Researchers have shown that changes in IDP concentration can affect assembly of RNP granules by concentrating different scaffold proteins and altering protein–protein interactions or protein–RNA interactions within RNP granules (Alberti & Hyman, 2016; Hyman et al., 2014). Some RBPs have different states under different protein concentrations. Concentration‐dependent LLPS requires a threshold protein concentration to drive the assembly of RNP granules. If a certain concentration is exceeded, however, the liquid‐like condensate is in danger of converting into a hydrogel‐ or solid‐like state. For instance, in vitro, at physiological concentrations, the RNA‐binding protein FUS separates into dynamic liquid compartments, but when the concentration is increased to higher levels, FUS converts into a gel‐like state. Over time, these intermediate hydrogel‐like compartments further transition into solid‐like fibrillar aggregates that no longer exchange materials with their surroundings (Molliex et al., 2015; Patel et al., 2015). These concentration‐induced changes in phase behavior could also trigger changes in the binding affinities of IDPs with their binding partners, leading to recruitment of distinct molecules into RNP granules. This in turn could lead to different RNP granule composition, physical properties, and structural organization (Alberti & Hyman, 2016).

Several lines of evidence indicate that aging is associated with the loss of ability to control gene expression and maintain protein homeostasis (Figure 1b). In the nematode *C. elegans*, extensive proteome remodeling and imbalances have been found to occur during aging, with a large amount of proteins increasing or decreasing in abundance, accompanied by widespread protein aggregation (Walther et al., 2015). Changes in protein concentration and impairment in the solubility of RNP granule‐forming proteins during aging would serve to impair SG formation or recruit different clients to RNP granules. Thus, the progressive failure of protein homeostasis that occurs during aging should impact SG assembly or disturb SG dynamic maintenance, which would lead to increased aggregation load.

In addition, during aging, cells are exposed to diverse types of stress, such as constant oxidative stress and declining acidity (Denoth Lippuner et al., 2014). Oxidative stress can induce the oxidation of proteins, lipids, and DNA, resulting in irreversible structural and functional damage (Gandhi & Abramov, 2012). Oxidative stress is also a known inducer of SG formation and a key factor in neurodegenerative disorders (Federico et al., 2012; Gandhi & Abramov, 2012; Patten, Germain, Kelly, & Slack, 2010). Acute oxidative stress promotes SG formation, and SGs disassemble when the stress is removed. However, persistent oxidative stress could trigger aggregation and oligomerization of some pathological RBPs such as TDP‐43, FUS, and tau (the most toxic species in neurodegenerative tauopathies), which could subsequently be recruited to SGs, serving to stabilize them (Chen & Liu, 2017; Lechler & David, 2017). Thus, it can be speculated that these aging‐associated chronic stress conditions could accelerate the formation of pathological aggregates of RBPs, causing additional functional defects and enhancing the nucleation of pathological SGs (Figure 1c), especially when the protein quality control system is overwhelmed.

It is worth noting that most of the conditions used to study SGs might have no or very little physiological relevance to aging and aging‐related diseases. For example, SGs can be induced by different oxidative stress reagents such as sodium arsenite and peroxide, but the constitutions of these SGs are different (Chen & Liu, 2017). This means that SGs induced by these reagents represent specific responses of cells to environmental changes, but these conditions do not necessarily mimic the intracellular state of aging cells. Also, when cells encounter acute stress, SGs can be induced and then removed after the insults subside, which is considered to be a protective measure of the cells. In contrast, during aging, persistent stress might induce continuous formation of SGs (Lechler & David, 2017), interfering with translation and the normal functions of important proteins trapped in the SGs. Thus, considering the differences between chronic stress and acute stress, more studies of SGs, including both their components and dynamics, should be performed under chronic stress conditions that mimic aging or disease‐related intracellular environmental changes.

Normal mitochondrial function, responsible for the production of the majority of the cell's ATP via oxidative phosphorylation (OXPHOS), is required for intracellular metabolism. Studies of mitochondrial morphology in different organisms reveal that mitochondrial structure and function change dramatically during aging (Hughes & Gottschling, 2012; Lam, Aung‐Htut, Lim, Yang, & Dawes, 2011; McFaline‐Fig ueroa et al., 2011; Scheckhuber et al., 2007; Veatch, McMurray, Nelson, & Gottschling, 2009). Further, mitochondrial dysfunction is known to impair energy production and normal metabolism. Recent studies have indicated that SG assembly, dynamics, disassembly, and clearance occur in an energy‐consuming manner (Buchan, Kolaitis, Taylor, & Parker, 2013; Cherkasov et al., 2013; Jain et al., 2016; Kroschwald et al., 2015; Loschi et al., 2009). Thus, although evidence indicating a direct link between mitochondrial dysfunction and SG dynamics is limited, it can be speculated that when cells are young, their active metabolism supports the assembly and healthy dynamics of SGs in the cell. However, with age, mitochondrial function declines, and this might lead to limited control of aberrant phase transition (Figure 1d). It is not clear whether defects in mitochondrial function and behavior are caused by aging or themselves contribute to the aging process and aging‐related degeneration. What is clear, however, is that mitochondrial dysfunctions have been linked to cancer, metabolic disorders, and neurodegenerative diseases, including Alzheimer's, Parkinson's, and Huntington's disease (Nunnari & Suomalainen, 2012). Thus, potential aberrant phase transitions induced by mitochondrial malfunction during aging might be a driver of neurodegenerative diseases.

#### 4.3.3 The influence of RBP mutations associated with aging‐related Neurodegenerative diseases on SG dynamics

RBP mutations that increase SG formation or limit SG clearance are known causative factors in some neurodegenerative diseases (Li, King, Shorter, & Gitler, 2013; Ramaswami, Taylor, & Parker, 2013). Many RBPs, such as FUS, TDP‐43, hnRNPA1, hnRNPA2B1, hnRNPDL, and TIA‐1, are typical components of RNP granules, and their missense mutations have been identified in inherited forms of ALS, FTD, and myopathy (Kim et al., 2013; Klar et al., 2013; Kwiatkowski et al., 2009; Prasad, Bharathi, Sivalingam, Girdhar, & Patel, 2019; Sreedharan et al., 2008; Vieira et al., 2014). Disease‐causing mutations in these proteins are associated with misfolding and aberrant mislocalization, resulting in disturbed SG assembly and clearance (Figure 1b). For instance, certain mutations in TAF15, EWSR1, hnRNPA1, and hnRNPA2/B1 accelerate misfolding and nucleation of SGs (Couthouis et al., 2011, 2012; Kim et al., 2013). ALS‐causing FUS and TDP‐43 mutations cause excessive cytoplasmic localization and misfolding (Bosco et al., 2010; Liu‐Yesucevitz et al., 2010; Vance et al., 2009), which might trigger dysregulated assembly of inappropriate fibrillar aggregates that are toxic (Johnson et al., 2009; Sun et al., 2011). Accelerated FUS and TDP‐43 aggregates alter the dynamics and physiological properties of RNP granules even after stress is removed (Parker et al., 2012), and this results in persistent SGs that have been observed in cells expressing ALS‐associated TDP‐43 or FUS mutants (Bosco et al., 2010; Liu‐Yesucevitz et al., 2010).

#### 4.3.4 Declining protein quality control system in aged cells impacts SG composition and dynamics

The protein quality control (PQC) system is an integrated network of molecular chaperones and two main degradative systems, which selectively degrade misfolded proteins and dysfunctional organelles, namely the ubiquitin–proteasome system (UPS) and autophagy, respectively (Amm, Sommer, & Wolf, 2014; Boya, Reggiori, & Codogno, 2013). When cells age, however, the PQC system and protein homeostasis are disrupted (Josefson, Andersson, & Nystrom, 2017). Chaperone expression levels and activity have been found to decrease in the brains of mice as they age, and this aging‐dependent decline contributes to the susceptibility of aged neuronal cells to misfolded proteins (Yang, Huang, Huang, Gaertig, Li, & Li, 2014). Further, analysis of chaperone expression in humans found that ATP‐dependent chaperone machines are repressed both in the aging human brain and in aging‐associated diseases (Figure 1e), indicating the importance of chaperones in aging and in prevention of the pathogenesis of neurodegenerative disease (Brehme et al., 2014). In addition, a large body of evidence supports an overall age‐dependent decrease in the amount of proteasomes or UPS subunits, as well as proteasomal activity, during the aging process (Figure 1e; Baraibar & Friguet, 2012; Bulteau, Szweda, & Friguet, 2002; Kastle & Grune, 2011; Saez & Vilchez, 2014).

Autophagy is a conserved PQC system involving the removal of damaged proteins and organelles in a lysosomal‐mediated pathway. Autophagic activity and lysosomes decline in an age‐dependent manner in muscles, heart, liver, intestine, pharynx, and neurons (Figure 1e; Chang, Kumsta, Hellman, Adams, & Hansen, 2017; Cuervo & Dice, 2000; Del Roso et al., 2003; Donati et al., 2001; Jiao & Demontis, 2017; Nixon, 2017; Yan & Finkel, 2017). Some evidence also indicates that disruption of neuronal autophagy results in neurodegeneration in animal models (Figure 1e; Hara et al., 2006; Komatsu et al., 2006; Zhao et al., 2013). Age‐related deterioration of autophagy, including chaperone‐mediated autophagy (CMA) and macroautophagy, is influenced by diverse factors. These include decreased autophagy‐related gene expression (Drummond et al., 2014; Glatigny et al., 2019; Joseph et al., 2013; Kiffin et al., 2007; McMullen, Ferry, Gamboa, Andrade, & Dupont‐Versteegden, 2009; Simonsen et al., 2008), decreased formation and clearance of autophagosomes (Stavoe, Gopal, Gubas, Tooze, & Holzbaur, 2019), sustained activation of mTORC1 signaling, which is a suppressor of autophagy (Bonaldo & Sandri, 2013), and lower protein levels of autophagy and SG core components (Moujaber et al., 2017), as well as mitophagy regulators (Russ, Boyd, McCoy, & McCorkle, 2015; Sebastian et al., 2016).

Severe stress‐ and aging‐related misfolded proteins could specifically accumulate and aggregate within SGs, which could alter SG composition, impair SG dynamics, and, finally, lead to aberrant conversion from a liquid‐like to a solid‐like state (Figure 1b). For example, under severe stress conditions like robust heat stress, stress granule components can interact with misfolded proteins via their PLDs, promoting the seeding of SGs (Kroschwald et al., 2015). Under mild stress conditions or normal growth conditions, the cellular chaperone machinery and degradation systems are sufficient to manage the surveillance of such aberrant interactions between RBPs and other aggregation‐prone proteins. During aging, however, PQC systems decline, resulting in compromised PQC systems that can be overrun, which might affect SG dynamics.

In support of this, researchers have found that inhibition of Hsp70 function in yeast and mammals leads to increased SG formation and delayed SG disassembly through an increase in the number of SGs containing misfolded proteins (Cherkasov et al., 2013; Mateju et al., 2017; Walters, Muhlrad, Garcia, & Parker, 2015). Ganassi and collaborators identified the HSPB8‐BAG3‐HSP70 chaperone complex as a key regulator of SG composition and dynamics. Once this chaperone‐mediated SG surveillance mechanism is disturbed, misfolded proteins and defective ribosomal products (DRiPs) accumulate in SGs, triggering an aberrant liquid‐to‐solid conversion with defective SG disassembly (Ganassi et al., 2016). The 26S proteasome can be recruited to SGs to promote their clearance (Turakhiya et al., 2018), and inhibition of the UPS induces SG formation, although this is not because of the failure of degradation of SG assembly factors (Mazroui, Marco, Kaufman, & Gallouzi, 2007).

In young cells, aberrant SGs can be cleared through another backup degradative system—autophagy—in which aberrant SGs are targeted to the vacuole. Inhibition of autophagy could affect SG clearance in mammalian cells, in which autophagic clearance of SG may be an important factor in reducing the pathology from various diseases (Buchan et al., 2013). However, in aged cells, impaired autophagy may be associated with enhanced SG formation and/or disturbed SG clearance, resulting in aberrant SG persistence after removal of the stressor (Figure 1e). This may be a cause of aging‐related neurodegenerative diseases. This is consistent with the association of reduced autophagy with accelerated aging, and the correlation between autophagy/ubiquitination failure and a wide range of disorders, including cancer and neurodegeneration (Saez & Vilchez, 2014; Yang & Klionsky, 2010). It may be due to that incompetence of the PQC systems results in the inability to eliminate aberrant SGs and protein aggregations in a timely manner (Madeo, Tavernarakis, & Kroemer, 2010; Rubinsztein, Marino, & Kroemer, 2011). In 2014, Seguin and colleagues found that autophagy, lysosomal activity, and VCP activity are not only involved in SG clearance, but also participate in proper SG assembly. Inhibition of their activities causes defective SG formation and alterations in SG morphology and composition (Seguin et al., 2014). This shows that autophagy can affect SG assembly and disassembly in a complicated process that requires further investigation.

Apart from this, less is known about how neuronal autophagy is regulated. In contrast to autophagy in non‐neuronal cells, which can be induced by starvation and other cellular stressors, autophagy in neurons is not significantly induced by such stressors (Maday, Wallace, & Holzbaur, 2012; Wong & Holzbaur, 2014). Also, although some progress has been made in revealing the relationship between autophagy and aging in non‐neuronal cells (Chang et al., 2017; Hansen, Rubinsztein, & Walker, 2018), little is known about whether these essential mechanisms work similarly in neurons. What we know is that disrupting autophagy in neurons has led to neurodegeneration in some animal models (Hara et al., 2006; Komatsu et al., 2006; Zhao et al., 2013). Recently, Stavoe and colleagues found that aging could affect autophagosome biogenesis and induce morphological changes of autophagic structures in neurons through regulation of the dynamics and phosphorylation state of WIPI2 (Stavoe et al., 2019; Stavoe & Holzbaur, 2020). In addition to initial autophagosome biogenesis, Nixon found that lysosomal integrity in later stages of autophagy also decreased with age in neurons (Nixon, 2017). Despite this, the regulation of other steps in neuronal autophagy, such as autophagosome closure, retrograde transport of autophagosomes, and cargo degradation, during aging is yet to be fully understood. Also not fully understood is how aging influences SG assembly and disassembly through a deficiency of neuronal autophagy.

## 5 POTENTIAL RELATIONSHIPS BETWEEN OTHER MEMBRANELESS ORGANELLES AND AGING OR AGING‐RELATED DISEASES

In addition to SGs, there are many other membraneless compartments that may be related to aging. These compartments differ in their composition, localization, size, and function. Examples of these non‐membrane‐bound assemblies include P bodies, P granules, neuronal transport granules, nucleoli, and Cajal bodies. Unlike SGs, which are enriched in translation initiation factors, P bodies are cytoplasmic RNA granules comprised primarily of the factors involved in mRNA degradation, translational repression, and RNA‐mediated gene silencing (Luo, Na, & Slavoff, 2018). Despite their differences, SGs and PBs also share many RNA‐binding proteins and RNA components, and are in close proximity and even overlap with each other (Buchan et al., 2008; Souquere et al., 2009). Given the existence of RNA decay factors in P bodies, they have been proposed to be involved in mRNA degradation and turnover. Currently, whether P bodies function in mRNA storage and/or mRNA decay remains actively debated (Luo et al., 2018; Standart & Weil, 2018). Combined with the finding that defects in RNA metabolism can trigger aging (Mazzoni & Falcone, 2011), to some extent these findings imply potential links between P bodies and aging. In addition, studies have indicated that P bodies are targeted for autophagy during the stationary phase, which suggests that aging‐related PQC declines might also affect P‐body dynamics (Buchan et al., 2013).

P granules are membraneless organelles composed of proteins and RNAs and found in the germline cytoplasm (Seydoux, 2018). They are thought to be sites of small RNA biogenesis and post‐transcriptional regulation, and are essential for the differentiation of germ cells into functional gametes during postembryonic development (Seydoux, 2018; Strome & Updike, 2015). Neuronal transport granules (or neuronal RNA granules) are motile granules that transport along microtubules to localize mRNAs and confer precise translational control during transport. They play important roles in neuronal development, maintenance, and activity (De Graeve & Besse, 2018; Kiebler & Bassell, 2006). As with SGs, mutations of some components of neuronal transport granules might induce hypo‐ or hyperassembly, further altering neuronal RNP granule function. Via this mechanism, they may be involved in neuropsychiatric disorders (De Graeve & Besse, 2018).

Cajal bodies and nucleoli are membraneless organelles in the nucleus. Cajal bodies, which contain coilin and survival motor neuron protein as well as other mRNA splicing factors, are thought to be the sites for assembly of small nuclear ribonuclear protein particles (snRNPs) (Banani et al., 2017; Buchan, 2014). The nucleoli are another example of RNA–protein complexes seeded by activation of rRNA transcription (Sleeman & Trinkle‐Mulcahy, 2014). Studies have found that, during chronic stress, alteration of physical conditions such as energy level changes affects the viscosity of non‐membrane‐bound organelles, including stress granules and nucleoli (Brangwynne et al., 2011).

Up to now, direct experimental evidence of the link between these nonmembrane assemblies and aging is still missing. Despite their diversity in unique molecular composition and corresponding functions, these membraneless compartments have several features in common. For example, Cajal bodies, nucleoli, P bodies, and P granules have been shown to have properties of liquid droplets and may form through liquid–liquid phase separation (Brangwynne et al., 2009, 2011; Kroschwald et al., 2015; Seydoux, 2018). As we know, that liquid–liquid phase separation process would be extremely sensitive to cellular environmental changes. The liquid‐like nature of these membraneless organelles makes them vulnerable to physical changes associated with aging, and they may be capable of quick conversion into more solid‐like structures. Thus, as with SGs, aging‐induced changes in metabolic activity and homeostatic capacity could dramatically shift other nonmembrane condensates’ phase behavior and physical properties. Specifically, some SG‐localized proteins, such as TDP‐43 and FUS, are also recruited to RNA transport granules and other non‐membrane‐bound organelles (Alami et al., 2014; Hennig et al., 2015; Mastrocola, Kim, Trinh, Rodenkirch, & Tibbetts, 2013). Thus, age‐related disease‐associated mutations of these proteins perhaps also affect the composition and dynamics of these membraneless compartments. This could partly explain why these membraneless assemblies are also associated with cancer and age‐related diseases (Alberti & Hyman, 2016; Sleeman & Trinkle‐Mulcahy, 2014).

## 6 CONCLUSION AND FUTURE PERSPECTIVES

An SG is a kind of stress‐inducible RNP granule formed by LLPS. SGs are sensitive to environmental changes and enable cells to be more flexible in response to these changes, facilitating their survival during stress. Any mechanisms that alter the local concentration, localization, and structural conformation of key SG components will influence the formation and dynamics of SGs, leading to the potential for an aberrant conversion into a pathogenic state (Banani et al., 2017). In young cells, multiple cellular defense systems can protect the cells from being affected by damaging changes such as imbalanced cellular proteostasis and proteolysis, inappropriate covalent modifications, and lowered pH levels. In aged cells, however, age‐dependent breakdown of such systems may lead to defects in maintaining normal SG assembly, dynamics, disassembly, and clearance. This in turn could lead to the subsequent onset of a barrage of diseases.

Despite research progress concerning the relationship between SGs and aging discussed in this review, further in‐depth investigations will help to reveal the mechanisms underlying the interactions between SGs and aging. First of all, what are the components of SGs formed under chronic stress caused by aging‐induced intracellular environmental changes? Dynamic analysis of changes in the properties of SGs and SG components during the aging process could provide vital clues on how aging influences SG formation. Whether these changes exert a synergistic effect that could accelerate aging will be an important question to be answered. Moreover, it is known that aggregation‐prone proteins can be recruited to SGs and that this could result in aberrant or persistent SGs during cellular stress and after the stress subsides. These aberrant SGs might induce a series of effects that can be attributed to reduced stress resistance with age. Such aberrant SGs may also act as seeds to facilitate the formation of irreversible mature protein aggregates in aged cells, further accelerating the decline of the cellular functions of these proteins. Thus, it seems that maintaining a proper SG dynamic might be a potential strategy to delay aging and increase lifespan. Two key questions that remain to be answered are as follows: (a) What kind of proteins are prone to form aggregates during aging? And (b) is aggregation triggered by interactions between aggregation‐prone proteins and SG components? Answers to these questions will have important implications for our understanding of the machineries underlying the relationship between SGs and aging. Key components identified from such studies might generate valuable pharmaceutical targets for the treatment of aging‐related diseases.

## CONFLICTS OF INTEREST

The authors have no conflicts of interest to disclose.

## AUTHOR CONTRIBUTIONS

XC and XJ wrote the manuscript with input from BL. XC, XJ, and BL contributed to the design of the manuscript. BL supervised the overall direction, planning, and writing of the manuscript.
